# Endoscopic lumbar foraminotomy for foraminal stenosis in stable spondylolisthesis

**DOI:** 10.3389/fsurg.2022.1042184

**Published:** 2022-11-10

**Authors:** Yong Ahn, Han Byeol Park, Byung Rhae Yoo, Tae Seok Jeong

**Affiliations:** Department of Neurosurgery, Gil Medical Center, Gachon University College of Medicine, Incheon, South Korea

**Keywords:** endoscopic, foraminal stenosis, foraminoplasty, foraminotomy, lumbar, percutaneous, spondylolisthesis

## Abstract

**Background:**

Open decompression with fusion is the gold-standard surgical technique for spondylolisthesis. However, it may be too extensive for patients with foraminal stenosis with stable spondylolisthesis. The endoscopic lumbar foraminotomy (ELF) technique was developed as a minimally invasive surgical option for foraminal stenosis. Some authors have reported the outcomes of ELF for various spondylolistheses. However, few studies have demonstrated foraminal stenosis in advanced stable spondylolisthesis. This study aimed to describe the surgical technique and results of ELF for radiculopathy due to foraminal stenosis in patients with stable spondylolisthesis.

**Methods:**

Consecutive 22 patients who suffered from radiculopathy with spondylolisthesis underwent ELF. The inclusion criterion was unilateral radicular leg pain due to foraminal stenosis in stable spondylolisthesis. After the percutaneous transforaminal approach, foraminal decompression was performed using various surgical devices under endoscopic visualization. Surgical outcomes were measured using the visual analog pain score, Oswestry disability index, and modified MacNab criteria.

**Results:**

Pain scores and functional outcomes improved significantly during the 12-month follow-up periods. The rate of clinical improvement was 95.5% (21 of 22 patients). One patient experienced a dural tear and subsequent open surgery.

**Conclusion:**

ELF can be effective in foraminal stenosis in stable spondylolisthesis. Technical points specializing in foraminal decompression in spondylolisthesis are required for clinical success.

## Introduction

The gold standard surgical technique for lumbar spondylolisthesis with foraminal stenosis is decompression and fusion surgery, which may be performed using different methods. However, this surgery may result in considerable morbidity or sequelae, particularly in older patients.

In cases of foraminal stenosis with fixed or stable spondylolisthesis, adequate foraminal decompression may be a good solution while avoiding the surgical risk of extensive fusion surgery. Therefore, a minimally invasive decompression technique is required for cases with stable stenosis.

The endoscopic lumbar foraminotomy (ELF) or foraminoplasty technique was developed for effective foraminal decompression under a working channel endoscopic view (1–4). The foraminal decompression technique has evolved using different surgical tools such as microforceps, lasers, bone trephines, and endoscopic burrs. Moreover, the advanced ELF technique is as effective as open foraminotomy (4). However, this technique is unfamiliar to standard spine surgeons and challenging for endoscopic surgeons.

Some studies have been published on transforaminal endoscopic decompression for spondylolisthesis with lumbar stenosis (5–12). However, most studies have described this technique for lumbar intracanal stenosis or disc herniation in spondylolisthesis. Furthermore, few studies have demonstrated transforaminal endoscopic decompression procedures specific to severe foraminal stenosis in patients with stable and advanced spondylolisthesis. Therefore, we believe this study will help aspiring endoscopic spine surgeons understand the endoscopic foraminal decompression procedure and apply this technique in exceptional cases such as spondylolisthesis.

This study aimed to demonstrate the clinical outcomes of ELF for foraminal stenosis in stable spondylolisthesis and describe a practical and technical approach to achieving good clinical outcomes with ELF.

## Materials and methods

### Patients and evaluation

Twenty-two consecutive patients with foraminal stenosis in spondylolisthesis were treated with ELF between January 2019 and January 2021. Cases were prospectively registered in the database, and records were retrospectively analyzed. The institutional review board approved the study, and written informed consent was obtained from all participants.

The inclusion criteria for ELF were as follows: 1) chronic unilateral radicular leg pain despite more than 3 months of nonoperative treatment, 2) foraminal stenosis in spondylolisthesis demonstrated on magnetic resonance imaging (MRI) and computed tomography (CT) scans, 3) spondylolisthesis without definitive hypermobility on dynamic x-rays, and 4) foraminal stenosis documented as the source of radiculopathy by imaging studies, neurologic examination, and selective nerve root block.

The exclusion criteria were low back pain alone, acute lumbar disc herniation, severe central stenosis, segmental instability or hypermobility, and other pathological conditions such as inflammation, infection, trauma, or tumor.

Changes in clinical status were assessed using the visual analog pain score (VAS) and Oswestry disability index (ODI). The global outcome was evaluated using the modified MacNab criteria. Follow-up data were obtained through regular outpatient clinic visits or telephone interviews.

### Surgical technique

The surgical procedure was performed according to a previously described method of ELF (4, 13). It consists of three processes: 1) the transforaminal approach under fluoroscopic view, 2) bone resection using endoscopic burrs, and 3) soft tissue removal using micropunches.

Intramuscular midazolam (0.05 mg/kg) and intravenous fentanyl (0.8 μg/kg) were administered on call. The patient was placed in a prone position on a radiolucent spine table.

#### Transforaminal approach (outside-in technique)

This step was performed to ensure the safe docking of the working sheath at the foraminal zone. The skin entry point and approach angle were determined according to the target point and body size on preoperative MRI, CT scan, and x-rays.

**Figure 1:**
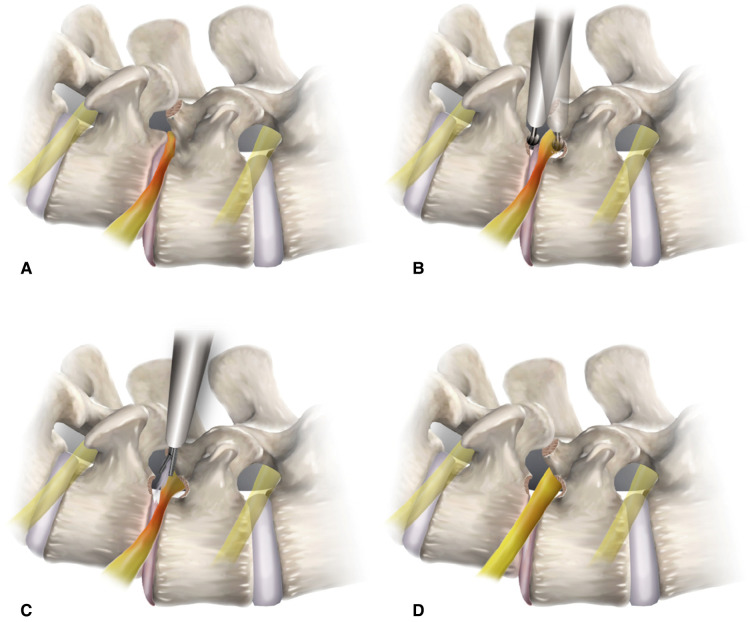
Conceptual illustrations depicting the surgical procedure of endoscopic lumbar foraminotomy for spondylolisthesis. **(A)** Foraminal docking of the working sheath viewing the foraminal surgical field protecting the exiting nerve root (outside-in approach). **(B)** Foraminal unroofing using endoscopic burrs for resecting the upper pedicle and lower vertebral endplate. **(C)** Soft tissue decompression with removal of the ligamentum flavum. **(D)** Final point of the full-scale foraminal decompression from the axillary side to the lateral exit zone.

**Figure 2:**
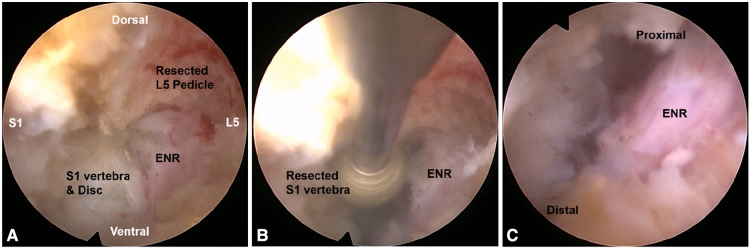
Intraoperative endoscopic views. Foraminal unroofing with the removal of the upper pedicle **(A)** and lower vertebral endplate **(B)** compressing the exiting nerve root (ENR). After the full-scale decompression, the ENR was freely released from the proximal axillary zone to the lateral exit zone **(C)**.

An 18-gauged needle was introduced into the foraminal zone in the posterolateral direction under fluoroscopic guidance (lateral and anteroposterior projections). The typical approach angle is approximately 45° for foraminal decompression and can be adjusted to become steeper when the pathologic point is located in the extraforaminal zone. The needle tip was deeply inserted into the foraminal disc or on the vertebral body, along the surface of the superior articular process (SAP). The needle was replaced with a guidewire, and an obturator was introduced along the guidewire until the head of the obturator was fitted into the foramen without any access pain. The beveled final working sheath was advanced along the obturator by gently tapping with a mallet and placed firmly in the foraminal zone with its sharp end away from the exiting nerve root (ENR). The surgical field was created outside the foramen, and decompression proceeded into the foramen (outside-in approach). Thus, the ENR was protected during the entire procedure (Figures 1A, 2A).

#### Endoscopic bone work

Endoscopic foraminal decompression was initiated after a working channel endoscope was inserted. The initial view included the ENR with perineural fat and disc surface. These structures helped the surgeon maintain the correct orientation during the entire procedure. Next, the surface of the SAP was exposed by rotating the working sheath and the endoscope. The tip of the SAP was then drilled using various endoscopic burrs along the ENR until the ligamentum flavum (LF) and foraminal ligaments at the axillary zone were sufficiently exposed. Finally, any bone or venous bleeding was coagulated using radiofrequency tips and hemostatic agents. In cases of advanced spondylolisthesis, the ENR is usually pinched by a narrow space between the upper pedicle and lower vertebral endplates rather than by the SAP. Therefore, the ENR should be decompressed by resecting these bony structures. Bone resection is an essential and critical process of foraminal decompression specific to spondylolisthesis cases (Figures 1B, 2B).

#### Endoscopic soft tissue work

**Figure 3:**
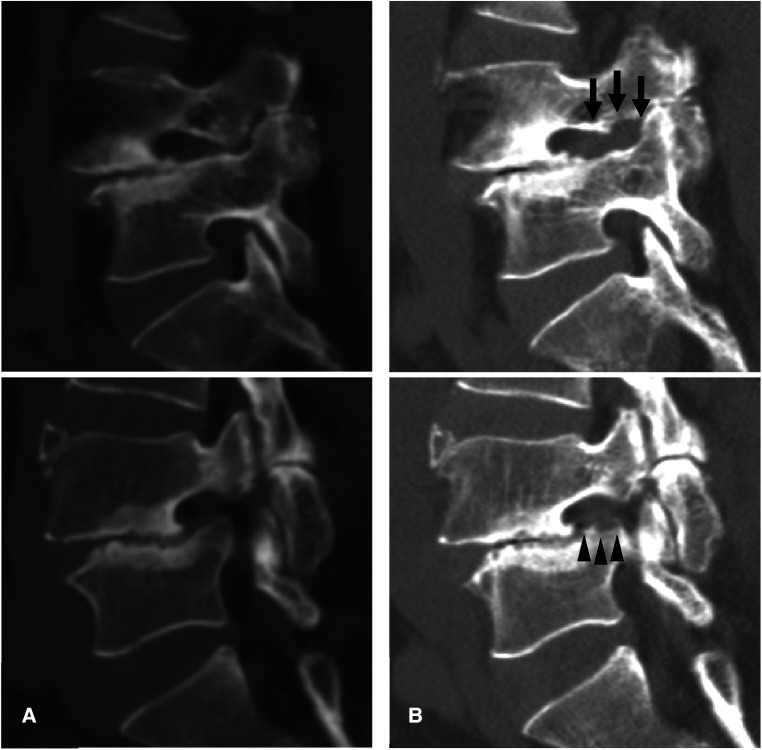
An illustrative case of a 62-year-old Male patient. **(A)** Preoperative computed tomography (CT) images showing foraminal stenosis with spondylolisthesis at the L4-5 level. **(B)** Postoperative CT images showing foraminal decompression with resection of a part of the upper pedicle (arrow) and lower vertebral endplate (arrowheads).

**Figure 4:**
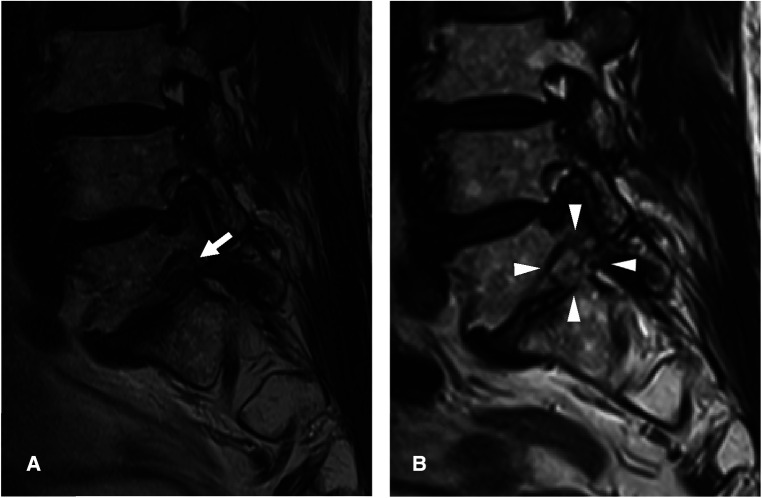
An illustrative case of a 75-year-old Male patient. **(A)** Preoperative magnetic resonance image (MRI) showing foraminal stenosis with spondylolisthesis at the L5-S1 level (arrow). **(B)** Postoperative MRI showing foraminal decompression with removal of the protruded disc and surrounding bony tissues (arrowheads).

After sufficient bone work, delicate soft tissue removal was performed, and the ENR was released. The decompression process was directed toward the proximal side, and the nerve root course was traced to the axillary epidural zone. The hypertrophied LF and protruding disc material were removed gradually using micropunches, forceps, and radiofrequency tips (Figure 1C). Although minor, bleeding may seriously interfere in the endoscopic surgical field. Therefore, meticulous hemostasis was essential to ensure a clear vision during the procedure. The ENR became exposed and released as soft tissue work proceeded. Surgeons were careful not to damage the dural membrane. The tissue debris was cleared with radiofrequency, and the neural tissues were separated from the offending tissues. The axillary epidural zone is a key landmark for foraminal decompression. Exposure of the dural sac to the starting point of the ENR indicated successful foraminal decompression. Once the proximal axillary zone was released, the nerve root was examined from the proximal side to the lateral exit zone. Any remaining ligament or disc tissue was trimmed during full-scale foraminal decompression. Finally, determining the definitive finishing point is mandatory to prevent an incomplete decompression. The endpoint of ELF was determined by sufficient exposure and strong pulsation of the neural tissue (Figures 1D, 2C). Postoperatively, the surgeon checked each patient's status for 3 h. The patient was discharged within 24 h in the absence of complications (Figures 3, 4).

### Statistical analysis

Statistical analysis was performed between the pre- and postoperative clinical results using repeated-measures analysis of variance and a paired t-test. Statistical significance was set at *P* < 0.05.

## Results

The mean age of the patients (14 females and 8 males) was 69.2 years (range, 53–83). The mean BMI was 22.94 ± 2.59 kg/m^2^. The degrees of spondylolisthesis were grade 1 in 20 patients (90.9%) and grade 2 in 2 (9.1%). The operating levels were L5-S1 in 12 (54.5%) patients, L4–5 in 8 (36.4%), and L3–4 in 2 (9.1%). The mean operative time was 63.6 min (range, 35–115). The mean postoperative hospital stay duration was 1.9 days (range, 1–5).

**Figure 5:**
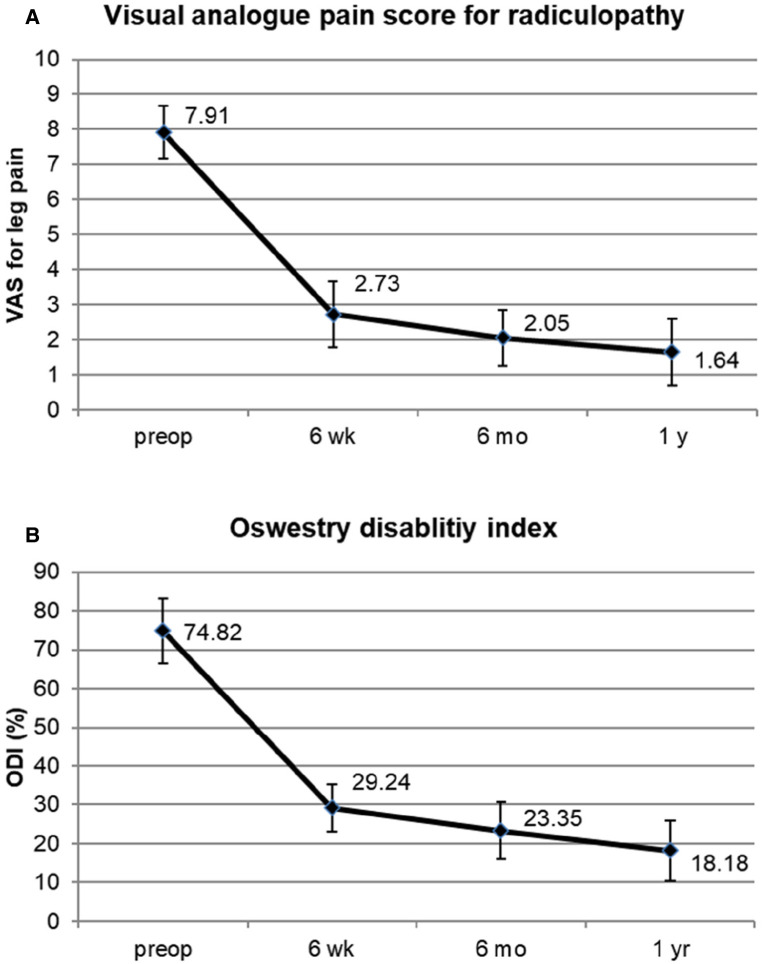
Clinical outcomes. **(A)** Visual analog pain score for radicular leg pain preoperatively and at 6 weeks, 6 months, and 1 year after surgery. **(B)** Oswestry disability index scores preoperatively and at 6 weeks, 6 months, and 1 year after surgery.

**Figure 6:**
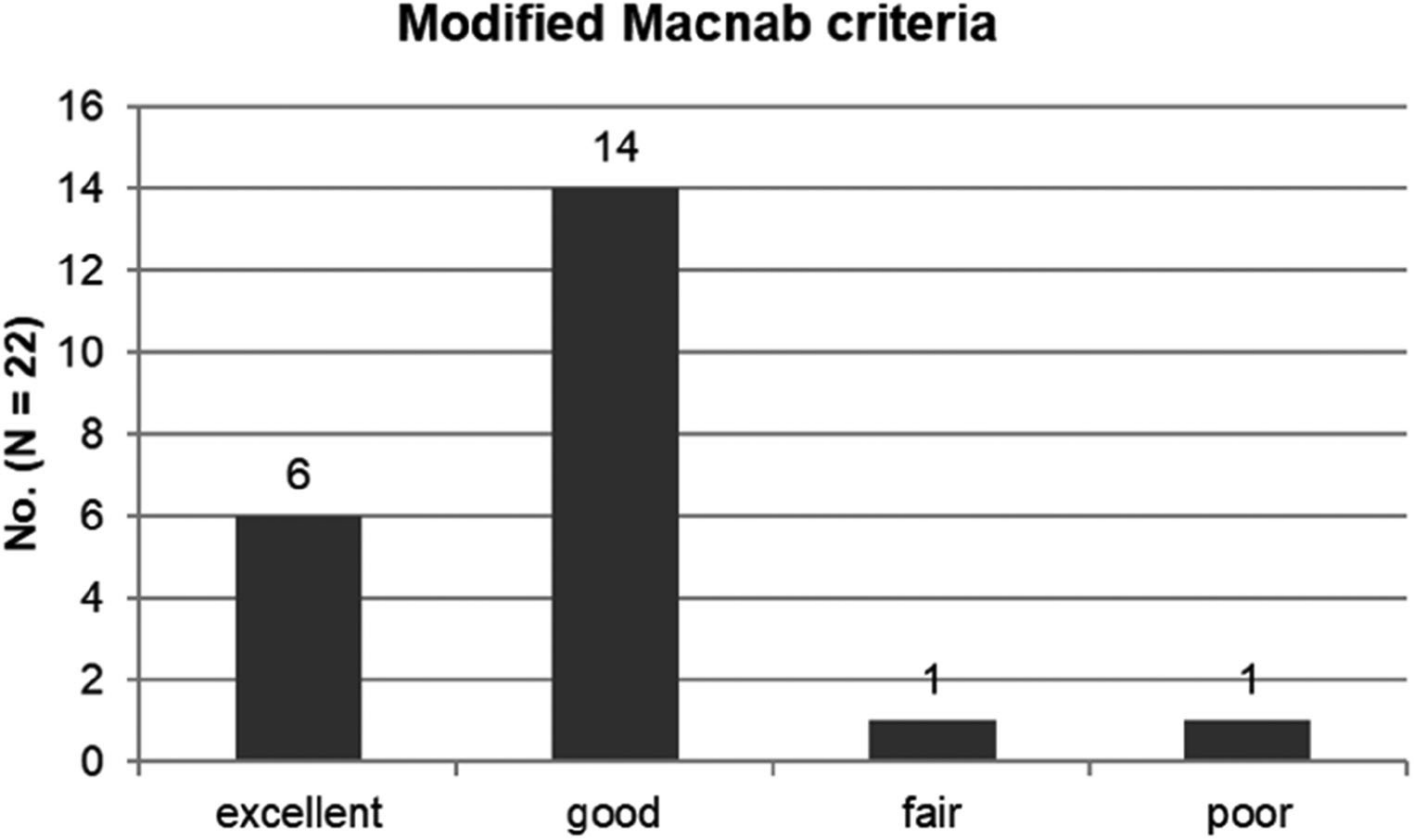
The global outcome according to the modified macNab criteria: excellent in 6 patients (27. 3%), good in 14 (63.6%), fair in 1 (4.5%), and poor in 1 (4.5%). Therefore, the success rate was 90.9%, and the clinical improvement rate was 95.5%.

The mean preoperative VAS score for the lumbar radiculopathy was 7.91 ± 0.75, which improved to 2.73 ± 0.94, 2.05 ± 0.79, and 1.64 ± 0.95 at 6 weeks, 6 months, and 1 year postoperatively, respectively (*P* < 0.001) (Figure 5A). The mean preoperative ODI was 74.82 ± 8.34%, which improved to 29.24 ± 6.08%, 23.35 ± 7.24%, and 18.18 ± 7.73% at 6 weeks, 6 months, and 1 year postoperatively, respectively (*P* < 0.001) (Figure 5B). The global results based on the modified MacNab criteria were rated as follows: excellent in 6 patients (27. 3%), good in 14 (63.6%), fair in 1 (4.5%), and poor in 1 (4.5%). Therefore, the success rate was 90.9%, and the clinical improvement rate was 95.5% (Figure 6).

During the procedure, one patient experienced a dural tear in the axillary zone at the L3–4 level. The patient complained of severe pain and underwent subsequent open surgery (transforaminal lumbar interbody fusion with dural repair). Otherwise, no other significant perioperative complications were observed. No newly developed back pain or radiological signs of further instability were noted during the follow-up period.

## Discussion

### Surgical data and clinical outcome

The ELF technique is usually suitable for geriatric patients because of its minimal invasiveness. However, the average age of the surgical candidates in this study was higher than that of other case series of ELF. The disease entity appears to be chronic radiculopathy due to long-standing or advanced spondylolisthesis. Therefore, most patients may be older individuals or long-suffering. Additionally, older patients do not prefer extensive fusion surgery for perioperative morbidities.

The operative data showed the typical benefits of minimally invasive spine surgery. The mean operative time was 63.6 min, which was shorter than that of open fusion surgery (14–17). Blood loss was negligible, and postoperative hospital stays were fairly straightforward. These findings can facilitate a patient's earlier return to ordinary life.

The patient outcomes significantly improved in both the VAS and ODI scores. The mean VAS score of radiculopathy decreased by 6.327 at the final evaluation (*P* < 0.001). Conversely, the mean ODI improved by 56.64 at the final assessment (*P* < 0.001). A reduction of more than 50% in the VAS score (18) or an improvement of more than 20%–30% in the ODI is clinically relevant (19, 20). Therefore, our data indicate that the ELF technique for spondylolisthesis is efficacious in ENR decompression and results in significant functional improvement.

The success rate (excellent or good) based on the modified MacNab criteria was 90.9%, with a clinical improvement rate of 95.5%. These findings are comparable to those of published open foraminotomy procedures (21–27).

Our series had no significant complications except for one dural tear and conversion to open surgery. None of the patients experienced any further clinical or radiological segmental instability during the follow-up period. Although some bony structures were removed to decompress the nerve root, the ELF technique did not cause the development of further instability in any of the patients in our study.

Given the innate characteristics of ELF, the clinical success and complication rates may depend on the surgeon's skill. However, once technical proficiency is achieved, surgeons can produce relevant and reliable results. Therefore, an extensive and systematic learning process is required to implement this procedure.

### History of ELF/foraminoplasty

Owing to the development of decompression devices, ELF has become a practical foraminal decompression technique. The first-generation procedure uses a laser for foraminal decompression. Knight et al. (1, 28) introduced an endoscopic laser foraminoplasty technique. The central concept of laser foraminoplasty is sculpting the foramen by ablating the hypertrophic foraminal ligaments using a side-firing laser under an endoscopic view. Although the soft tissues and fibrotic adhesion could evaporate, the hard tissue or hypertrophic bone could not be effectively removed with the laser beam. The second-generation technique uses bone trephine or reamer. Ahn et al. (2) reported an endoscopic foraminotomy technique using a bone trephine and Ho: YAG side-firing lasers. Schubert and Hoogland (29) described a foraminoplasty method using a bone trephine to remove the migrated lumbar disc herniation. Being a blind percutaneous technique under fluoroscopic view, the use of bone trephine has inherent limitations, such as possible bone bleeding and neural injury. The ELF procedure employed in this study was achieved with the third-generation technique, in which spine surgeons applied endoscopic burrs and punches. Specially designed surgical tools enable precise, full-scale foraminal decompression as effective as open foraminotomy (1, 30–32).

### Current studies and theoretical benefits

Since Knight et al. published endoscopic lumbar laser foraminoplasty for isthmic spondylolisthesis (5), some authors have published transforaminal endoscopic decompression techniques for lumbar stenosis or disc herniation in spondylolisthesis (6–12). They decompressed the spinal canal or herniated disc using various surgical devices, such as lasers, trephines, forceps, and burrs. However, few studies have described precise techniques specific to foraminal stenosis in stable and advanced spondylolisthesis. Moreover, in stable spondylolisthesis, open decompression and fusion surgery under general anesthesia may be too extensive in foraminal stenosis without intracanalicular stenosis.

Without open fusion surgery, ELF can resolve chronic and intractable radiculopathy caused by spondylolisthesis. In addition, this minimally invasive technique may be efficient for patients who refuse fusion surgery or medically compromised older patients because the procedure can be performed percutaneously under local anesthesia. Consequently, the surgical complications of extensive fusion surgery can be reduced, and the patient can return to normal life earlier.

However, this minimally invasive procedure has a steep learning curve and limited indications. Therefore, the clinical application of ELF in spondylolisthesis should be carefully considered.

### Technical keys specific to foraminal stenosis with spondylolisthesis

Hypertrophic SAP and thickened LF compressing the ENR are the primary pathologies of foraminal stenosis. Therefore, the basic ELF technique consists of bone resection of the SAP and removal of the LF by endoscopic burrs and other surgical devices. The final landmark of the decompression process is the axillary epidural space, which is the starting point of the ENR.

However, the foraminal anatomy of advanced spondylolisthesis is different. Unlike the usual foraminal stenosis cases, the main offending structure may be the lower vertebral endplate rather than the SAP. In the foraminal zone of spondylolisthesis, the SAP is away from the ENR because of slippage of the upper vertebral body. Therefore, the ENR may impinge between the upper pedicle and lower vertebral body.

To achieve sufficient foraminal decompression, the surgeon should target the lower vertebral endplate rather than the SAP during the initial approach. Once the working sheath and endoscope are ensured to be in the foraminal working zone, the surgeon should confirm the route of the ENR and disc between the upper pedicle and lower vertebral endplate. Next, the upper pedicle and lower vertebral endplate should be sculptured using an endoscopic burr and punch. Finally, the ENR is released between the two resected bony walls after bone work.

### Limitation of the study

This study had some limitations. First, the study was conducted retrospectively without a control group. Therefore, selection bias in the inclusion criteria may have been present. Therefore, a prospective randomized trial or comparative cohort study comparing ELF and open fusion surgery for foraminal stenosis with spondylolisthesis is warranted. Second, the one-year follow-up period may be relatively short for drawing a conclusive result because the spondylolisthesis status or segmental stability may change with time, even after successful decompression. Therefore, a long-term follow-up study with a larger number of cases is required to verify the effectiveness of ELF for foraminal stenosis in spondylolisthesis.

## Conclusion

The advanced ELF technique is effective in adequately selected cases of lumbar spondylolisthesis. In addition, ELF may be suitable for intractable radiculopathy due to foraminal stenosis with fixed spondylolisthesis without segmental hypermobility—a specialized technique is required for the clinical success of foraminal decompression in spondylolisthesis. Moreover, it may provide an excellent minimally invasive alternative to extensive fusion surgery in older or medically compromised patients.

## Data Availability

The raw data supporting the conclusions of this article will be made available by the authors, without undue reservation.

## References

[1] Knight MT Vajda A Jakab GV Awan S. Endoscopic laser foraminoplasty on the lumbar spine–early experience. Minim Invasive Neurosurg. (1998) 41:5–9. 10.1055/s-2008-1052006 9565957

[2] Ahn Y Lee SH Park WM Lee HY. Posterolateral percutaneous endoscopic lumbar foraminotomy for L5-S1 foraminal or lateral exit zone stenosis. Technical note. J Neurosurg. (2003) 99(3 Suppl):320–3. 10.3171/spi.2003.99.3.0320 14563153

[3] Yeung AT. The evolution and advancement of endoscopic foraminal surgery: one surgeon's Experience incorporating adjunctive techologies. SAS J. (2007) 1:108–17. 10.1016/SASJ-2006-0014-RR 25802587 PMC4365579

[4] Ahn Y Oh HK Kim H Lee SH Lee HN. Percutaneous endoscopic lumbar foraminotomy: an advanced surgical technique and clinical outcomes. Neurosurgery. (2014) 75:124–33. 10.1227/NEU.0000000000000361 24691470 PMC4086756

[5] Knight M Goswami A. Management of isthmic spondylolisthesis with posterolateral endoscopic foraminal decompression. Spine (Phila Pa 1976) (2003) 28:573–81. 10.1097/01.BRS.0000050400.16499.ED 12642765

[6] Jasper GP Francisco GM Telfeian AE. Transforaminal endoscopic discectomy with foraminoplasty for the treatment of spondylolisthesis. Pain Physician. (2014) 17:E703–8. 25415785

[7] Yeung A Kotheeranurak V. Transforaminal endoscopic decompression of the lumbar spine for stable isthmic spondylolisthesis as the least invasive surgical treatment using the YESS surgery technique. Int J Spine Surg. (2018) 12:408–14. 10.14444/5048 30276099 PMC6159720

[8] Yamashita K Tezuka F Manabe H Morimoto M Hayashi F Takata Y Successful endoscopic surgery for L5 radiculopathy caused by far-lateral disc herniation at L5-S1 and L5 isthmic grade 2 spondylolisthesis in a professional baseball player. Int J Spine Surg. (2018) 12:624–8. 10.14444/5077 30364859 PMC6198622

[9] Li XF Jin LY Lv ZD Su XJ Wang K Song XX Endoscopic ventral decompression for spinal stenosis with degenerative spondylolisthesis by partially removing posterosuperior margin underneath the slipping vertebral body: technical note and outcome evaluation. World Neurosurg. (2019) 126:e517–25. 10.1016/j.wneu.2019.02.083 30825627

[10] Liu K Kadimcherla P. Transforaminal endoscopic lumbar decompression for isthmic spondylolisthesis: technique description and clinical outcome. Surg Technol Int. (2020) 36:467–70. 32227330

[11] Telfeian AE Syed S Oyelese A Fridley J Gokaslan ZL. Endoscopic surgical resection of the retropulsed S1 vertebral endplate in L5-S1 spondylolisthesis: case series. Pain Physician. (2020) 23:E629–36. 33185381

[12] Wu Q Yuan S Fan N Du P Li J Yang L Clinical outcomes of percutaneous endoscopic lumbar discectomy for the treatment of grade I and grade II degenerative lumbar spondylolisthesis: a retrospective study with a minimum five-year follow-up. Pain Physician. (2021) 24:E1291–8. 34793656

[13] Ahn Y Lee SG. Percutaneous endoscopic lumbar foraminotomy: how I do it. Acta Neurochir (Wien). (2022) 164:933–6. 10.1007/s00701-022-05114-z 35020086

[14] Cheng JS Park P Le H Reisner L Chou D Mummaneni PV. Short-term and long-term outcomes of minimally invasive and open transforaminal lumbar interbody fusions: is there a difference? Neurosurg Focus. (2013) 35:E6. 10.3171/2013.5.FOCUS1377 23905957

[15] Phan K Rao PJ Kam AC Mobbs RJ. Minimally invasive versus open transforaminal lumbar interbody fusion for treatment of degenerative lumbar disease: systematic review and meta-analysis. Eur Spine J. (2015) 24:1017–30. 10.1007/s00586-015-3903-4 25813010

[16] Hey HW Hee HT. Open and minimally invasive transforaminal lumbar interbody fusion: comparison of intermediate results and complications. Asian Spine J. (2015) 9:185–93. 10.4184/asj.2015.9.2.185 25901228 PMC4404531

[17] Lee MJ Mok J Patel P. Transforaminal lumbar interbody fusion: traditional open versus minimally invasive techniques. J Am Acad Orthop Surg. (2018) 26:124–31. 10.5435/JAAOS-D-15-00756 29337717

[18] Martin WJ Ashton-James CE Skorpil NE Heymans MW Forouzanfar T. What constitutes a clinically important pain reduction in patients after third molar surgery? Pain Res Manag. (2013) 18:319–22. 10.1155/2013/742468 23957018 PMC3917796

[19] Ng LC Tafazal S Sell P. The effect of duration of symptoms on standard outcome measures in the surgical treatment of spinal stenosis. Eur Spine J. (2007) 16:199–206. 10.1007/s00586-006-0078-z 16496190 PMC2200689

[20] Ostelo RW Deyo RA Stratford P Waddell G Croft P Von Korff M Interpreting change scores for pain and functional status in low back pain: towards international consensus regarding minimal important change. Spine (Phila Pa 1976). (2008) 33:90–4. 10.1097/BRS.0b013e31815e3a10 18165753

[21] Kunogi J Hasue M. Diagnosis and operative treatment of intraforaminal and extraforaminal nerve root compression. Spine (Phila Pa 1976). (1991) 16:1312–20. 10.1097/00007632-199111000-00012 1750006

[22] Donaldson WF 3rd Star MJ Thorne RP. Surgical treatment for the far lateral herniated lumbar disc. Spine (Phila Pa 1976). (1993) 18:1263–7. 10.1097/00007632-199308000-00003 8211356

[23] Lejeune JP Hladky JP Cotten A Vinchon M Christiaens JL. Foraminal lumbar disc herniation. Experience with 83 patients. Spine (Phila Pa 1976). (1994) 19:1905–8. 10.1097/00007632-199409000-00007 7997922

[24] Darden BV 2nd Wade JF Alexander R Wood KE Rhyne AL 3rd Hicks JR. Far lateral disc herniations treated by microscopic fragment excision. Techniques and results. Spine (Phila Pa 1976). (1995) 20:1500–5. 10.1097/00007632-199507000-00011 8623070

[25] Baba H Uchida K Maezawa Y Furusawa N Okumura Y Imura S. Microsurgical nerve root canal widening without fusion for lumbosacral intervertebral foraminal stenosis: technical notes and early results. Spinal Cord. (1996) 34:644–50. 10.1038/sc.1996.116 8918959

[26] Hodges SD Humphreys SC Eck JC Covington LA. The surgical treatment of far lateral L3-L4 and L4-L5 disc herniations. A modified technique and outcomes analysis of 25 patients. Spine (Phila Pa 1976). (1999) 24:1243–6. 10.1097/00007632-199906150-00012 10382252

[27] Chang HS Zidan I Fujisawa N Matsui T. Microsurgical posterolateral transmuscular approach for lumbar foraminal stenosis. J Spinal Disord Tech. (2011) 24:302–7. 10.1097/BSD.0b013e3181f7cc9f 20975597

[28] Knight MT Goswami A Patko JT Buxton N. Endoscopic foraminoplasty: a prospective study on 250 consecutive patients with independent evaluation. J Clin Laser Med Surg. (2001) 19:73–81. 10.1089/104454701750285395 11443793

[29] Schubert M Hoogland T. Endoscopic transforaminal nucleotomy with foraminoplasty for lumbar disk herniation. Oper Orthop Traumatol. (2005) 17:641–61. 10.1007/s00064-005-1156-9 16369758

[30] Sairyo K Chikawa T Nagamachi A. State-of-the-art transforaminal percutaneous endoscopic lumbar surgery under local anesthesia: Discectomy, foraminoplasty, and ventral facetectomy. J Orthop Sci. (2018) 23:229–36. 10.1016/j.jos.2017.10.015 29248305

[31] Ahn Y. Percutaneous endoscopic decompression for lumbar spinal stenosis. Expert Rev Med Devices. (2014) 11:605–16. 10.1586/17434440.2014.940314 25033889

[32] Yeung A Gore S. Endoscopic foraminal decompression for failed back surgery syndrome under local anesthesia. Int J Spine Surg. (2014) 8:22. 10.14444/1022 25694939 PMC4325507

